# Novel PKC-ζ to p47^phox^ interaction is necessary for transformation from blebbishields

**DOI:** 10.1038/srep23965

**Published:** 2016-04-04

**Authors:** Goodwin G. Jinesh, Rikiya Taoka, Qiang Zhang, Siddharth Gorantla, Ashish M. Kamat

**Affiliations:** 1 Department of Urology, Unit 1373, The University of Texas MD Anderson Cancer Center, 1515 Holcombe Boulevard, Houston, Texas, 77030, USA

## Abstract

Cancer stem cells are capable of transformation after apoptosis through the blebbishield emergency program. Reactive oxygen species (ROS) play an essential role in transformation. Understanding how ROS are linked to blebbishield-mediated transformation is necessary to develop efficient therapeutics that target the resurrection of cancer stem cells. Here we demonstrate that a novel PKC-ζ to p47^phox^ interaction is required for ROS production in cancer cells. The combined use of the S6K inhibitor BI-D1870 with TNF-α inhibited the PKC-ζ to p47^phox^ interaction, inhibited ROS production, degraded PKC-ζ, and activated caspases-3 and -8 to block transformation from blebbishields. BI-D1870 also inhibited transformation from cycloheximide-generated blebbishields. Thus ROS and the PKC-ζ to p47^phox^ interaction are valid therapeutic targets to block transformation from blebbishields.

Bladder cancer is classified broadly into non-muscle invasive and muscle invasive subtypes for therapeutic purposes^1^. Approximately 80% of patients with bladder cancer have non-muscle invasive bladder cancer at the time of diagnosis^2^. Non-muscle invasive bladder cancer is treated primarily with BCG immunotherapy^3^, which works predominantly by recruiting neutrophils into the bladder^1^. Neutrophils in turn secrete cytotoxic cytokines such as TNF-α upon BCG stimulation^4^,^5^. Although many patients respond to BCG immunotherapy, a significant proportion of patients exhibit BCG resistance, indicating that the cancer cells have managed to overcome the cytotoxic signal exerted by TNF-α^4^,^5^. Often cancer stem cells are implicated in such therapy resistance^6^,^7^, necessitating the need for combination therapeutics that can sensitize the cancer stem cells to TNF-α–induced apoptosis. In line with this view, we had previously identified Smac mimetics as an efficient therapeutic agent when combined with TNF-α^5^. Recently we found that cancer stem cells can resurrect after apoptosis by blebbishield emergency program–mediated sphere formation (transformation)^8^,^9^,^10^,^11^. ROS have been implicated in both induction of apoptosis and cellular transformation^12^,^13^,^14^,^15^. However, the role of ROS in cancer stem cells exhibiting blebbishield-mediated transformation is not understood. Examining the role of ROS in blebbishield-mediated transformation will help to identify how cancer stem cells resist cell death and help to find more combination therapeutics that can be used with BCG immunotherapy for non-muscle invasive bladder cancer.

Here we show that RT4P bladder cancer cells mount efficient ROS production at the 3-hour time point upon serum withdrawal and that the combination of the known apoptosis-inducing Smac mimetic plus TNF-α eliminates this ROS production. Using this ROS elimination as a criterion, we found that an S6K inhibitor, BI-D1870, efficiently eliminated ROS in combination with TNF-α in the absence of serum and efficiently induced apoptosis in the presence of serum. We further identified that PKC-ζ interacts with the regulatory component of the ROS-generating machinery, p47^phox^. Combined TNF-α and BI-D1870 efficiently disrupted the PKC-ζ to p47^phox^ interaction, resulting in the induction of apoptosis. Furthermore, BI-D1870 effectively blocked transformation from blebbishields. Thus ROS and the PKC-ζ to p47^phox^ interaction are effective therapeutic targets to block transformation from blebbishields, and BI-D1870 may lay a platform to develop novel combination therapeutics to BCG immunotherapy.

## Results

### Serum starvation induces robust ROS production and is blocked by a known apoptosis-inducing Smac mimetic combination with TNF-α

**Figure 1:**
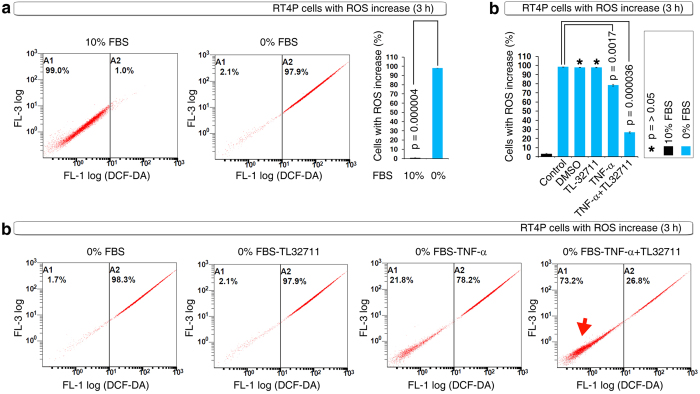
ROS play a survival role in RT4P cells. **(a)** RT4P cells mount efficient ROS production at the 3-hour time point of serum withdrawal, as shown by DCF-DA FACS (ROS FACS). **(b)** DCF-DA FACS showing that TNF-α and its combination with the Smac mimetic TL-32711 inhibits ROS production at 3 hours of serum withdrawal (top panel); reversal of cells back to ground state ROS (arrow) by TNF-α and/or its combination with TL-32711 is shown in dot plots (bottom panel; only critical panels were shown).

ROS are involved in the induction of apoptosis^13^ and are implicated in the process of cellular transformation in response to professional oncogenes such as K-Ras^15^. Although apoptosis and cellular transformation are two processes with divergent outcomes (i.e., cell death and survival), our recent findings demonstrated that cancer stem cells can undergo cellular transformation (sphere formation) after apoptosis^3^. Thus, ROS could play a critical role in the resurrection of cancer stem cells after apoptosis. Hence we first sought to determine whether ROS plays a role towards survival or cell death in RT4P bladder cancer cells (the bladder cancer cell line in which the blebbishield emergency program was discovered^8^). Serum withdrawal resulted in a tremendous increase in ROS-positive cells at the 3-hour time point (Fig. 1a). This ROS increase is a survival response because a known apoptosis-inducing and transformation inhibiting combination (TNF-α plus the Smac mimetic TL-32711^5^; TL-32711 was previously known as compound-C^5^) significantly inhibited serum withdrawal-induced ROS-positive cell numbers (Fig. 1b). These data demonstrated that ROS production is a survival response in RT4P cells.

### S6K inhibitor BID-1870 blocks serum starvation-induced ROS either alone or more efficiently with TNF-α

**Figure 2:**
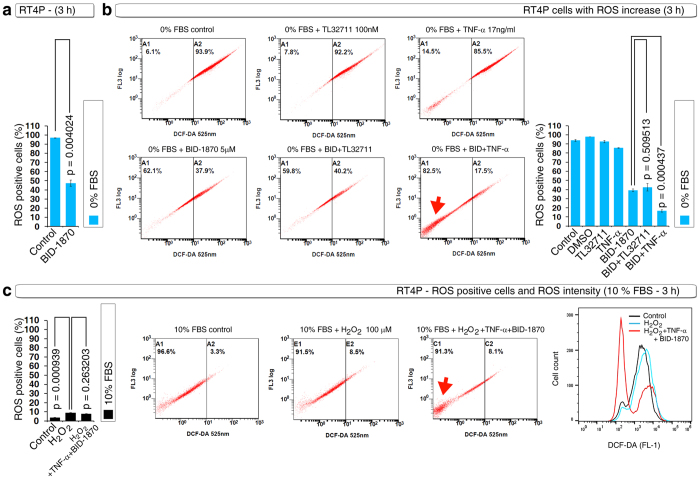
The S6K inhibitor BI-D1870 effectively blocks serum withdrawal–induced ROS with TNF-α but not with the Smac mimetic. **(a)** DCF-DA FACS showing that the S6K inhibitor BI-D1870 (5 μM) inhibits serum withdrawal–induced ROS as a single agent. **(b)** RT4P cells were treated with BID-1870 (5 μM) with or without TNF-α or TL-32711 for 3 hours in serum-free MEM and subjected to DCF-DA FACS. Note that BI-D1870 effectively inhibits ROS with TNF-α (arrow) but not with TL-32711. **(c)** RT4P cells were treated with BID-1870 (5 μM) plus TNF-α with or without 100 μM H_2_O_2_ for 3 hours in 10% FBS containing MEM and subjected to DCF-DA FACS. The intensity peaks were merged with Flowjo.

Using this ROS inhibition assay as a criterion, we tested several compounds (data not shown) and found the S6K inhibitor BI-D1870 significantly inhibited serum withdrawal-induced ROS-positive cell numbers (Fig. 2a). We further examined whether BI-D1870 could enhance the inhibition of serum withdrawal-induced ROS-positive cell numbers with TNF-α or TL-32711 and found that BI-D1870 significantly enhanced TNF-α–mediated block in ROS-positive cell numbers (Fig. 2b). We next tested whether H_2_O_2_ can induce increase in ROS positive cell number in the presence of serum. H_2_O_2_ slightly increased ROS positive cell numbers in the presence of serum but BI-D1870 and TNF-α combination drastically reduced the ROS intensity in the presence of H_2_O_2_ and serum (Fig. 2c). These data indicated that BI-D1870 could induce apoptosis in RT4P cells.

### Single-agent BID-1870 induces apoptosis in ROS^low^ cells

**Figure 3:**
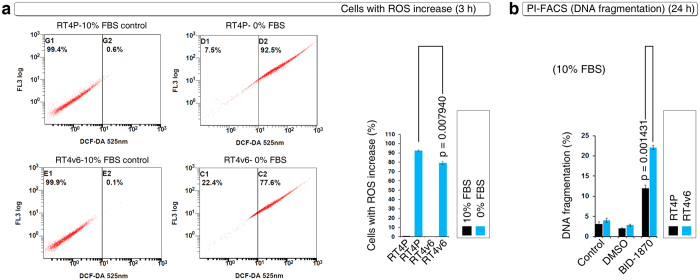
RT4v6 an efficient tumorigenic cell line than its parental RT4 cells (RT4P) exhibits lower ROS production but increased sensitivity to BI-D1870. (**a**) DCF-DA FACS showing lower ROS production in RT4v6 cells than in RT4P cells in response to serum withdrawal. Note the approximately 10% difference in ROS increased cells (bar graph). (**b**) DNA fragmentation analysis using PI-FACS in the presence of 10% serum shows that BI-D1870 (5 μM) as single agent induces increased DNA fragmentation in RT4v6 cells compared to RT4P cells. Note the approximate 10% increase in DNA fragmentation.

We next examined the serum withdrawal-induced ROS-positive cell numbers in RT4P cells and in its increased tumorigenic version, RT4v6 cells^8^. RT4v6 cells had significantly lower number of ROS-positive cells compared to RT4P cells in response to serum withdrawal at 3 hours (Fig. 3a). Single-agent treatments revealed that RT4v6 cells were significantly sensitive to BI-D1870 and this correlated with the low ROS-positive cell number in response to serum withdrawal (Fig. 3b compared with Fig. 3a). These data indicated that BI-D1870 induces apoptosis in low ROS-positive cells or ROS confers resistance to single agent BI-D1870.

### BID-1870 induces robust apoptosis with TNF-α in the presence of serum

**Figure 4:**
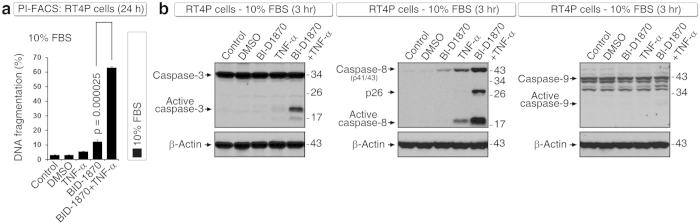
BI-D1870 induces robust apoptosis with TNF-α in RT4P cells. **(a)** DNA fragmentation analysis using PI-FACS in the presence of 10% serum shows that BI-D1870 (5 μM) in combination with TNF-α induces robust DNA fragmentation at 24 hours. **(b)** In the presence of 10% serum, BI-D1870 (5 μM) in combination with TNF-α induces robust caspase-3 and caspase-8 but not caspase-9 activation at the 3-hour time point, as analyzed by Western blotting. Note the p26 generation in caspase-8 (Asp-391 cleaved) in the combination condition (see text for details).

Although prolonged serum withdrawal can induce apoptosis in cancer cells due to stress^16^, inducing apoptosis in the presence of serum is appropriate for therapeutic candidates because serum starvation of cancer cells is not feasible in patients. In the presence of serum, TNF-α in combination with BI-D1870 induced robust DNA fragmentation at 24 hours (Fig. 4a) and induced caspase-3 and caspase-8 activation at the 3-hour time point (Fig. 4b). However, caspase-9 activation was not evident at 3 hours (Fig. 4b) or at 24 hours (data not shown), which suggested that caspase-3 and caspase-8 activations at 3-hour time points are early events and might be related to inhibition of ROS. Of note, generation of the caspase-8 p26 fragment was specifically observed under conditions of combined TNF-α plus BI-D1870 treatment (Fig. 4b). This result is very important because we had observed similar caspase-8 p26 fragment generation specifically during induction of apoptosis with BCG-stimulated neutrophil conditioned medium in combination with Smac mimetic^5^, indicating that the apoptosis thus induced is relevant to BCG immunotherapy.

### BID-1870 in combination with TNF-α targets novel PKC-ζ to p47^phox^ interaction and PKC-ζ expression

**Figure 5:**
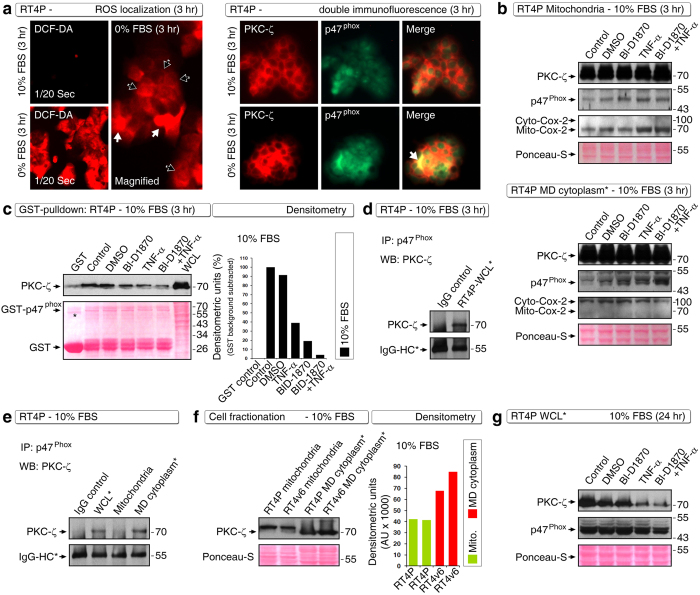
BI-D1870 in combination with TNF-α targets a novel PKC-ζ interaction with p47^phox^. **(a)** Localization of ROS in RT4P cells. DCF-DA fluorescence was imaged using a constant exposure of 1/20^th^ second. Note the fluorescence increases upon serum withdrawal. ROS was localized throughout the cells in a fraction of cells (white arrows), whereas in many cells ROS was localized in the cytoplasm (black arrows) (left panel). Double immunofluorescence data showing that PKC-ζ is partially colocalized with p47^phox^ in RT4P cells and this co-localization was increased upon serum withdrawal (arrow) (right panel). Note the predominant localization of PKC-ζ in the cytoplasm. **(b)** RT4P cell fractionation and Western blotting to show the localization of PKC-ζ and p47^phox^ in both mitochondria (top panel) and mitochondria-depleted cytoplasm (MD cytoplasm*) (bottom panel). Cytoplasmic Cox-2 and mitochondrial Cox-2 were analyzed to show the purity of fractionation. Note the increased localization of p47^phox^ in the mitochondrial-depleted cytoplasm under the BI-D1870 combination with TNF-α (correlating with the immunofluorescence data). **(c)** Full-length GST-p47^phox^ pulldown showing that the PKC-ζ interaction with p47^phox^ is targeted by BI-D1870 combination with TNF-α (left panel), with its quantitation by densitometry (right panel). Note: for densitometry, the GST background is subtracted from densitometry values. *Nonspecific band. **(d)** Immunoprecipitation to validate the interaction of PKC-ζ with p47^phox^ in RT4P whole-cell lysates. **(e)** Immunoprecipitation showing the interaction of PKC-ζ with p47^phox^ in mitochondria-depleted cytoplasm*. WCL*, whole cell lysate. **(f)** Western blot showing increased expression of PKC-ζ at RT4v6 mitochondria-depleted cytoplasm* (left) and its quantification by densitometry (right). **(g)** Western blot showing degradation of PKC-ζ in RT4P cells at the 24-hour time point.

We next examined how ROS is related to survival and how the BI-D1870 plus TNF-α combination overcome it. We first evaluated the sites of ROS production by DCF-DA fluorescence microscopy and found that ROS production was increased tremendously upon serum withdrawal (Fig. 5a). Upon serum starvation for 3 hours, ROS was predominantly localized at the cytoplasm and in few cells it was localized throughout the cells (Fig. 5a), suggesting that the ROS might be produced at cytoplasm in majority of the cells. We next attempted to identify the molecular basis of the ROS production and survival role. For this purpose, we examined PKC-ζ, and p47^phox^ component of the NOX organizer (ROS-producing machinery) because PKC-ζ is known to regulate p47^phox^ by phosphorylation^17^,^18^. We first examined the localization of PKC-ζ and p47^phox^ by double immunofluorescence and found that PKC-ζ was predominantly localized in the cytoplasm and p47^phox^ was predominantly localized in the nucleus of RT4P cells at basal levels in 10% FBS-containing medium (Fig. 5a). However, upon serum withdrawal for 3 hours, we observed co-localization of PKC-ζ and p47^phox^ at cytoplasmic area (Fig. 5a).

We next examined what happens to PKC-ζ and p47^phox^ localization under conditions of BI-D1870 plus TNF-α treatment in the presence of serum at the 3-hour time point. Biochemical characterization of PKC-ζ and p47^phox^ localization in mitochondria and mitochondria-depleted cytoplasm showed an increased accumulation of p47^phox^ in mitochondria-depleted cytoplasm in response to TNF-α plus BI-D1870 treatment (Fig. 5b). Interestingly, there was no such increase of p47^phox^ in mitochondria even though both PKC-ζ and p47^phox^ were present in mitochondrial fractions (Fig. 5b). Because PKC-ζ is known to phosphorylate p47^phox^, we examined whether these two molecules interact in RT4P cells. PKC-ζ interacted with p47^phox^ under basal conditions in the presence of serum (IMEx/IntAct accession number: 24704); however, this interaction was inhibited by BI-D1870 or TNF-α and even more effectively by their combination at the 3-hour time point, as assessed by full-length GST-p47^phox^-pulldown assay (Fig. 5c). This result explains why TNF-α and BI-D1870 had an inhibitory action on ROS production. In line with this finding, PKC-ζ, p47^phox^ and ROS are known to have an altruistic feedback loop in which PKC-ζ activation is required for NADPH-dependent (p47^phox^ is a NADPH component) ROS production and ROS production leads to sustained PKC-ζ activation^19^. Thus inhibition of the PKC-ζ to p47^phox^ interaction is an important target for TNF-α–induced apoptosis in combination with BI-D1870. We further validated this interaction using immunoprecipitation and found that PKC-ζ co-immunoprecipitated with endogenous p47^phox^ under basal conditions in the presence of serum (Fig. 5d).

Next we examined the location of this interaction by fractionating cytoplasm into mitochondria and mitochondria-depleted cytoplasm and found that the PKC-ζ to p47^phox^ interaction occurs in the cytosol but not in mitochondria (Fig. 5e). Because RT4v6 cells had significantly fewer ROS-positive cells than did RT4P cells (Fig. 3a), we examined whether these cells differ in PKC-ζ expression in the cytosol. As expected, RT4v6 cells had increased PKC-ζ localization to mitochondria-depleted cytosol but expressed similar quantities of PKC-ζ in the mitochondria (Fig. 5f).

Examination of PKC-ζ and p47^phox^ at the 24-hour time point demonstrated that PKC-ζ but not p47^phox^ was degraded by the BI-D1870 plus TNF-α combination (Fig. 5g). It should be noted that the PKC-ζ to p47^phox^ interaction was inhibited even in the presence of adequate PKC-ζ expression in mitochondria-depleted cytosol at the 3-hour time point, demonstrating that the inhibition of interaction is not due to PKC-ζ degradation at this time point (Fig. 5b compared with Fig. 5c). Hence the loss of interaction between PKC-ζ and p47^phox^ could be an early event that triggers the loss of ROS production at 3 hour time point and which in turn may result in the loss of PKC-ζ activity and its degradation.

### PKC-ζ expression and its interaction with p47^phox^ are necessary for transformation from blebbishields

**Figure 6:**
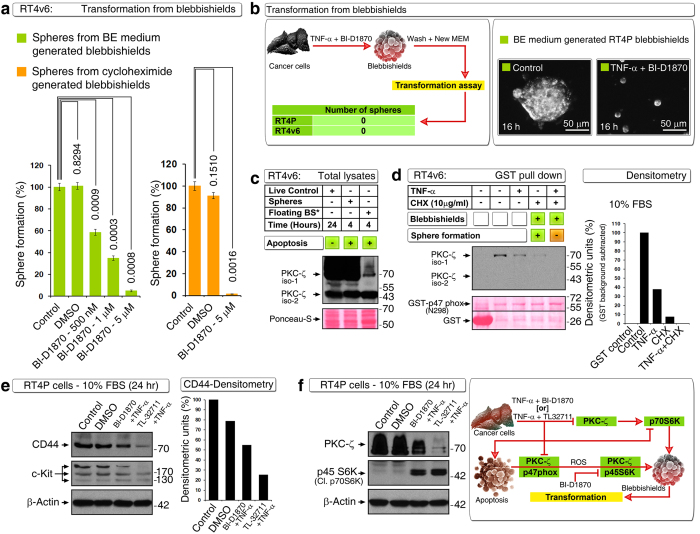
PKC-ζ expression and its interaction with p47^phox^ is required for transformation from blebbishields. **(a)** BI-D1870 inhibits transformation from blebbishields generated from RT4v6 cells using BE medium (left) or using cycloheximide (right). **(b)** Blebbishields generated from RT4P and RT4v6 cells using TNF-α plus BI-D1870 (5 μM) are not able to transform in complete MEM (left panel). Photomicrograph showing that transformation from BE medium generated blebbishields is completely blocked by TNF-α plus BI-D1870 (5 μM) in complete MEM (right panel). **(c)** PKC-ζ isoform-1 expression is required for transformation from blebbishields. Note that the floating/non-sphere-forming blebbishields (floating BS*) express very low levels of PKC-ζ compared to sphere-forming blebbishields (spheres) (see text for details). **(d)** GST pulldown showing that N-terminal 298 amino acids of p47^phox^ are sufficient to interact with PKC-ζ and that this interaction is retained in CHX generated blebbishields (lane 4) and is lost in CHX plus TNF-α generated blebbishields (lane 5) (left); see quantification by densitometry (right). Note: GST background is subtracted from the densitometric values. **(e)** Downregulation of CD44 or c-Kit may not be crucial to block transformation as TNF-α plus BI-D1870 (5 μM) did not downregulate stem cell markers as efficiently as TNF-α plus TL32711 (100 nM) (left WB panel): CD44 quantified by densitometry (right panel). **(f)** Downregulation of PKC-ζ and generation of cleaved p70S6K (p45S6K) are common to both combinations (TNF-α plus BI-D1870 and TNF-α plus TL32711) (left panel): schematic showing the links between PKC-ζ, p47^phox^, ROS, p45S6K (see text for references) (right panel).

We next examined whether BI-D1870 can inhibit transformation from blebbishields. For this purpose, we generated blebbishields using blebbishield ejection (BE) medium (BE medium: 1 μM cisplatin with 20 mM lithium chloride in 10% FBS containing MEM that lacks vitamins and amino acids) from RT4v6 cells (blebbishields from RT4v6 cells are known to undergo more efficient transformation than the blebbishields from RT4P cells^8^). BI-D1870 inhibited transformation from BE medium generated blebbishields in a dose-dependent manner with maximal inhibition at 5 μM concentration (Fig. 6a). Furthermore, we generated blebbishields using 10 μg/ml cycloheximide (CHX) in complete MEM for 24 hours and found that BI-D1870 at 5 μM concentration was able to inhibit transformation from blebbishields efficiently (Fig. 6a). We next generated blebbishields using the TNF-α and BI-D1870 (5 μM) combination and plated the blebbishields in complete MEM without any inhibitors for transformation. Interestingly, blebbishields generated from RT4P and RT4v6 cells using TNF-α plus BI-D1870 (5 μM) combination completely lost the ability to undergo transformation (Fig. 6b left panel). Furthermore, TNF-α plus BI-D1870 (5 μM) combination could block transformation from BE medium generated blebbishields in the presence of serum (Fig. 6b right panel).

We next examined the role of PKC-ζ in transformation from blebbishields. For this purpose, we generated blebbishields from RT4v6 cells using 10 μg/ml CHX in complete MEM for 24 hours and allowed the blebbishields to form spheres for 4 hours in complete MEM before isolating floating non-sphere-forming blebbishields and attached sphere-forming blebbishields. Analysis of total lysates of sphere-forming blebbishields and non-sphere-forming blebbishields by Western blotting indicated that PKC-ζ was significantly lost in non-sphere-forming blebbishields but not in sphere-forming blebbishields, demonstrating the requirement of PKC-ζ for transformation from blebbishields (Fig. 6c). Of note, the PKC-ζ isoform-1 is more important for transformation than its isoform-2, because both sphere-forming blebbishields and non-sphere-forming blebbishields expressed similar quantities of PKC-ζ isoform-2 (Fig. 6c).

We next examined whether PKC-ζ isoform-1 interaction with p47^phox^ is required for transformation from blebbishields. For this purpose, we used CHX and combined TNF-α plus CHX in RT4v6 cells to generate blebbishields, where a sub-set of blebbishields generated from CHX are capable of transformation but the blebbishields from TNF-α plus CHX are not^20^. Under these conditions, we tested the interaction of PKC-ζ with N-terminal 298 amino acids of p47^phox^ fused to GST as bait and found that the PKC-ζ interaction with GST- p47^phox^ N-terminal 298 amino acids was retained in CHX generated blebbishields (despite being reduced significantly due to the proportion of non-sphere-forming blebbishields also present among CHX generated blebbishields), whereas this interaction was abolished in TNF-α plus CHX generated blebbishields (Fig. 6d). Next we examined stem cell markers CD44 and c-Kit and found that TNF-α plus BI-D1870 moderately reduced CD44 and c-Kit expression despite TNF-α plus TL32711 had a greater effect in reduction (Fig. 6e). Recently we reported that PKC-ζ and full length/cleaved p70S6K/p45S6K have important roles in blebbishield mediated transformation^9^,^10^. Both TL32711 and BI-D1870 induced reduction in expression of PKC-ζ and generated p45S6K with TNF-α (Fig. 6f), indicating PKC-ζ and p70S6K are crucial targets that are common to both combinations. Interestingly, p70S6K is a downstream target of PKC-ζ^21^. Taken together, these results demonstrate that PKC-ζ expression and its interaction with p47^phox^ are required for transformation from blebbishields and that the N-terminal 298 amino acids of p47^phox^ are sufficient to interact with PKC-ζ.

## Discussion

BCG immunotherapy is known to work through the induction of functional TNF-α from neutrophils^4^,^5^. This fact enabled us to rapidly screen combination therapeutics that can be used along with BCG immunotherapy by simply using TNF-α as a surrogate. We previously demonstrated that the Smac mimetic compound-C (currently known as TL-32711 or birinapant) efficiently induced apoptosis with TNF-α^5^. In this study, we identified BI-D1870 as an efficient combination agent that can be coupled with TNF-α and thus with BCG immunotherapy in the future to abrogate blebbishield-mediated resurrection of cancer stem cells after apoptosis.

Identification of the novel PKC-ζ to p47^phox^ interaction (IMEx/IntAct accession number: 24704) is a crucial aspect, because loss of this interaction results in inhibition of ROS production, and this interaction is abolished under conditions that are not permissive to transformation from blebbishields but retained under conditions that are permissive to transformation (Fig. 6d).

Despite the fact that ROS are known to induce apoptosis, accumulating evidence shows that ROS is necessary for cellular transformation^15^,^22^. In this context, the altruistic feedback loop of PKC-ζ, p47^phox^, and ROS gains importance as PKC-ζ activates the NADPH component p47^phox^ to generate ROS, and ROS in turn offer sustained activation status for PKC-ζ^19^. Both PKC-ζ and ROS has vital role in blebbishield biology. PKC-ζ interacts with serpentine filopodia forming machinery PAK-1 along with cdc42 to construct blebbishields from apoptotic bodies^9^. On the other hand Smac mimetic in combination with TNF-α, blocks ROS production to block transformation from blebbishields^20^. Thus ROS play an essential role in cellular transformation. In support of this view, PKC isoforms have been implicated in K-Ras and ROS-mediated cellular transformation^15^. Significantly reduced PKC-ζ expression in non-sphere-forming blebbishields, but not in sphere-forming blebbishields, underscores the requirement of PKC-ζ expression.

Increased sensitivity of RT4v6 cells compared with RT4P cells to single-agent BI-D1870 indicates that RT4v6 cells depend on S6K more than RT4P cells do (Fig. 3). We had previously shown that RT4v6 blebbishields express more full-length p70S6K than RT4P blebbishields do and that RT4v6 cells exhibited increased transformation from blebbishields^8^. It should also be noted that RT4v6 cells have relatively fewer ROS-positive cells than do RT4P cells (Fig. 3). However, ROS are required for transformation^15^,^20^,^23^. These facts indicate that ROS positivity in non-apoptotic conditions is distinct from ROS positivity during apoptosis and thus in blebbishields. For example, IGFBP-3 is known to increase ROS to induce apoptosis^24^ and also suppress ROS to promote tumor growth^25^. It is possible that ROS-induced apoptosis could lead to transformation after apoptosis and hence ROS-forming machinery should be shut down during apoptosis. In support of this notion, we have previously shown that ROS inhibition during blebbishield formation is necessary to block transformation from blebbishields^20^.

Although TNF-α or BI-D1870 as single agent could significantly block ROS generation, neither could induce significant apoptosis as single agent, indicating that TNF-α and BI-D1870 act in a synergistic manner to inhibit ROS production and to induce apoptosis (Fig. 2 compared with Fig. 4). Often combination treatments target cells that are competent for transformation despite not being able to induce apoptosis in an entire population of cells^26^.

In summary, our study identified a novel combination of TNF-α with BI-D1870 that targets a novel PKC-ζ to p47^phox^ interaction and degrades PKC-ζ to block the ROS-mediated survival advantage, resulting in the inhibition of transformation from blebbishields.

## Methods

### Cells and cell maintenance

Human bladder cancer cells RT4 (ATCC-HTB-2; referred to as RT4 parental/RT4P in this study) and RT4v6 (a serially xenograft-passaged, cancer stem cell–enriched version of RT4P cells) were cultured in MEM with 10% fetal bovine serum, L-glutamine, pyruvate, non-essential amino acids, vitamins, penicillin, and streptomycin. For some experiments, the cells were cultured in serum-free MEM for 3 hours as indicated in the figures.

### Reagents and antibodies

TL-32711 (A-1901; used at 100 nM throughout) was purchased from Active Biochem (Maplewood, NJ, USA), BI-D1870 (S2843) from Selleckchem (Houston, TX, USA), and TNF-α (210-TA; used at 17 ng/ml throughout) from R&D Systems (Minneapolis, MN, USA). LiCl2 (L-4408), H_2_O_2_ (1009) and DCF-DA (2′,7′-dichlorofluorescein diacetate: D6883) were purchased from Sigma (St. Louis, MO, USA). Cycloheximide (239763) was purchased from Calbiochem (San Diego, CA, USA), cisplatin (NDC 0015-3220-22) from Bristol Laboratories (Princeton, NJ, USA), and protein-A sepharose CL-4B (17-0780-01) from GE Healthcare (Pittsburgh, PA, USA). Antibodies to active caspase-3 (Sc-7148), caspase-9 (Sc-7885), PKC-ζ (Sc-17781), and p47^phox^ (Sc-14015) were purchased from Santa Cruz Biotechnology (Santa Cruz, CA, USA). Antibody to caspase-8-Asp-391 (9496) was purchased from Cell Signaling Technology (Beverly, MA, USA).

### ROS quantification by FACS and imaging

RT4P or RT4v6 cells were plated at a density of 50,000 cells/ml and 4 ml/well onto six-well plates. Twenty-four hours later, cells were treated as indicated in the figures for 23.5 or 2.5 hours. At this point, 10 μM DCF-DA was added to all wells, and cells were incubated for a further 30 minutes before analysis of ROS-activated DCF-DA fluorescence (FL-1/525 nm) by FACS (Beckman Coulter, FC500)^27^,^28^.

Alternatively, the cells were imaged after DCF-DA addition using fluorescence microscopy (Olympus) with 1/20 second constant exposure between conditions (DP manager/controller).

### Double immunofluorescence

RT4P cells were plated onto 24-well plates at the density of 50,000 cells/ml and at 24 hours of plating the medium was changed to serum free or complete MEM and incubated for further 3-hours. The medium was then aspirated out and immediately fixed using ice-cold methanol at −20 °C for more than 24 hours. The cells were blocked in 1% BSA in PBS with 0.3% Triton X-100 for 30 minutes at 4 °C. PKC-ζ (1:50 in blocking buffer) and p47^phox^ (1:50 in blocking buffer) antibodies were incubated with cells in blocking buffer with Triton X-100 for 16 hours at 4 °C. Cells were washed three times in PBS and incubated further with Alexa-555 and Alexa-488 conjugated secondary antibodies (1:1500 dilution in blocking buffer) for 1 hour at room temperature in the dark. The cells were then washed three times with PBS before proceeding to fluorescence microscopy.

### Isolation of mitochondria and mitochondria-depleted cytoplasm

The cells were treated as indicated in the figures and were scraped in the medium, pelleted, and washed with ice-cold PBS at 1000 × g for 5 minutes at each step. Mitochondria were isolated as previously described^29^. The cytosolic fractions obtained as byproducts were clarified at 13,000 rpm for 10 minutes and saved. Isolated mitochondria were lysed in lysis buffer (as described in the Western blotting section) for 40 minutes, clarified at 13,000 rpm, quantified using the BCA assay, and subjected to Western blotting.

### SDS-PAGE, Western Immunoblotting, and densitometry

The cells/blebbishields were washed with PBS and resuspended in lysis buffer [50 mM Tris-HCL (pH 7.4), 150 mM NaCl, 5 mM EDTA, 25 mM NaF, 1% Triton X-100, 1% NP-40, 0.1 mM Na_3_VO_4_, 12.5 mM β-glycerophosphate, 1 mM PMSF, and complete protease inhibitor cocktail (Roche)] and incubated in ice for 40 minutes with vortexing for 20 seconds every 10 minutes (a total of 40 minutes increases the extraction of proteins from mitochondria and nuclei). The lysates were clarified at 13,000 rpm for 10 minutes, and the supernatants were quantified using the BCA assay reagent (Pierce). The lysates were subjected to SDS-PAGE and Western blotting on nitrocellulose membranes. The membranes were developed using ECL reagent (GE Healthcare). For selected blots, densitometry was performed using ImageJ software^30^.

### Immunoprecipitation

Antibody to p47^phox^ and control antibody (2 μg/reaction) were conjugated to protein-A sepharose CL4B beads for 30 minutes at 4 °C in a rotating platform before being washed three times (500 rpm for 30 seconds at 4 °C) with whole-cell lysis buffer (composition described in the Western immunoblotting section) and then incubated with 200 μg of RT4P whole-cell lysates/mitochondrial lysates/mitochondria-depleted cytoplasm (diluted to a total of 400 μl in whole-cell lysis buffer) for 2.5 hours at 4 °C. Immunoprecipitates were washed three times with whole-cell lysis buffer before being subjected to SDS-PAGE and Western immunoblotting for PKC-ζ.

### Plasmids and GST-pulldown assays

DH5α strain bacteria were transformed with pGEX constructs and induced with 1 mM IPTG (I6758; Sigma) for 4 hours at either 37 °C (for GST and p47^phox^ N-terminal 298–amino acid construct^31^, a kind gift from Dr. Lance S. Terada, University of Texas Southwestern Medical Center, Dallas, TX, USA) and GST control construct or at 30 °C (for p47phox full-length constructs^15^,^31^, kind gifts from Dr. Lance S. Terada and Dr. S-J Lee, Research Institute for Natural Sciences, Hanyang University, Seoul, Korea). They were then lysed using sonication (output: 8; 15-second bursts, twice in ice) in bacteria lysis buffer (20 mM HEPES [pH 7.5], 120 mM NaCl, 10% (v/v) glycerol, 2 mM EDTA, 1 mM DTT, 0.5% NP-40, 1x- protease inhibitor cocktail, and 1 mM PMSF), clarified at 13,000 rpm for 10 minutes, and bound to glutathione sepharose-CL4B for 1 hour at 4 °C. The beads were then washed six times using bacteria lysis buffer and incubated with 200 μg/200 μl cancer cell lysates (as indicated in the figures) prepared in whole-cell lysis buffer (composition described in the Western blotting section) +200 μl of magnesium-containing lysis buffer (25 mM HEPES [pH 7.5], 150 mM NaCl, 1% (v/v) NP-40, 0.25% (w/v) sodium deoxycholate, 10% glycerol, 20 mM MgCl_2_, 1 mM EDTA, 1x- protease inhibitor cocktail, and 1 mM PMSF) for 1 hour at 4 °C. Pulldown complexes were washed four times with magnesium-containing lysis buffer before being subjected to SDS-PAGE and immunoblotting.

### Protein interaction database submission

The protein interactions from this publication have been submitted to the International Molecular Exchange Consortium (IMEx Consortium) (http://www.imexconsortium.org) through IntAct^32^ and assigned the identifier IM-24704.

### Blebbishield-mediated transformation (sphere formation) assays

Blebbishield-mediated transformation (sphere-formation) assays were performed in RT4P and RT4v6 cells.

For the transformation shown in Fig. 6a, blebbishields were isolated after treating RT4v6 cells using 10 μg/ml CHX treatment or using BE medium (MEM with 1 μM cisplatin and 20 mM freshly prepared LiCl_2_ as described previously^8^) for 24 hours. The floating blebbishields were isolated, washed (1200 rpm for 3 minutes), and allowed to form spheres in six-well plates in the presence or absence of BI-D1870 in complete MEM for 16–24 hours as indicated in the figures. The floating non-sphere-forming blebbishields were washed off using complete MEM, and the attached spheres were covered with MEM and counted by scanning the well from end to end.

For the transformation shown in Fig. 6b, RT4P and RT4v6 cells were treated with TNF-α plus BI-D1870 (5 μM) for 24 hours to induce apoptosis and blebbishield formation. The floating blebbishields were then collected, pelleted at 1200 rpm for 3 minutes, washed, and plated in complete MEM for 16–24 hours before the floating blebbishields were washed off and scanning the wells for sphere count.

### Isolation of sphere-forming and non-sphere-forming blebbishields

Sphere-forming and non-sphere-forming blebbishields were isolated as described previously^20^. Briefly, RT4v6 cells were plated at a density of 200,000 cells/ml (13 mL/T-75 flask × 4 flasks) and at 24 hours were treated with 10 μg/ml CHX for further 24 hours. The floating pyknotic cell populations were then gently collected, pelleted down at 1200 rpm for 3 minutes at room temperature, washed once with complete MEM, and re-plated in 150-mm plates with complete MEM for a further 4 hours. The floating cells (non-sphere-forming blebbishields) were washed with complete MEM and collected separately, and the attached spheres (sphere-forming blebbishields) were scraped into fresh ice-cold complete MEM. Both types of blebbishields were pelleted at 3500 rpm for 5 minutes at 4 °C and washed with ice-cold PBS before lysis and Western blotting.

### Statistical analyses

Statistical analyses were performed using Microsoft Excel 2010. The statistical significance was determined based on Student’s *t*-test with two-tailed distribution and two-sample unequal variance, and *P* values < 0.05 were considered significant. Error bars represent standard errors (SEM).

## Additional Information

**How to cite this article**: Jinesh, G. G. *et al.* Novel PKC-ζ to p47^phox^ interaction is necessary for transformation from blebbishields. *Sci. Rep.* **6**, 23965; doi: 10.1038/srep23965 (2016).
